# Comprehensive Proteomic Analysis of Lysine Acetylation in the Foodborne Pathogen *Trichinella spiralis*

**DOI:** 10.3389/fmicb.2017.02674

**Published:** 2018-01-11

**Authors:** Yong Yang, Mingwei Tong, Xue Bai, Xiaolei Liu, Xuepeng Cai, Xuenong Luo, Peihao Zhang, Wei Cai, Isabelle Vallée, Yonghua Zhou, Mingyuan Liu

**Affiliations:** ^1^Key Laboratory of Zoonosis Research, Ministry of Education, Institute of Zoonosis/College of Veterinary Medicine, Jilin University, Changchun, China; ^2^Wu Xi Medical School, Jiangnan University, Affiliated Hospital of Jiangnan University, Wuxi, China; ^3^State Key Laboratory for Molecular Biology of Special Economic Animals, Institute of Special Economic Animal and Plant Sciences, Chinese Academy of Agricultural Sciences, Changchun, China; ^4^China Institute of Veterinary Drug Control, Beijing, China; ^5^State Key Laboratory of Veterinary Etiological Biology, Key Laboratory of Veterinary Parasitology of Gansu Province, Lanzhou Veterinary Research Institute, Chinese Academy of Agricultural Sciences, Lanzhou, China; ^6^JRU BIPAR, ANSES, École Nationale Vétérinaire d’Alfort, INRA, Université Paris-Est, Animal Health Laboratory, Maisons-Alfort, France; ^7^Jiangsu Institute of Parasitic Disease, Wuxi, China

**Keywords:** lysine acetylation, post-translational modification, lysine acetylation motif, interaction network, *Trichinella spiralis*

## Abstract

Lysine acetylation is a dynamic and highly conserved post-translational modification that plays a critical role in regulating diverse cellular processes. *Trichinella spiralis* is a foodborne parasite with a considerable socio-economic impact. However, to date, little is known regarding the role of lysine acetylation in this parasitic nematode. In this study, we utilized a proteomic approach involving anti-acetyl lysine-based enrichment and highly sensitive mass spectrometry to identify the global acetylated proteome and investigate lysine acetylation in *T. spiralis*. In total, 3872 lysine modification sites were identified in 1592 proteins that are involved in a wide variety of biological processes. Consistent with the results of previous studies, a large number of the acetylated proteins appear to be involved in metabolic and biosynthetic processes. Interestingly, according to the functional enrichment analysis, 29 acetylated proteins were associated with phagocytosis, suggesting an important role of lysine acetylation in this process. Among the identified proteins, 15 putative acetylation motifs were detected. The presence of serine downstream of the lysine acetylation site was commonly observed in the regions surrounding the sites. Moreover, protein interaction network analysis revealed that various interactions are regulated by protein acetylation. These data represent the first report of the acetylome of *T. spiralis* and provide an important resource for further explorations of the role of lysine acetylation in this foodborne pathogen.

## Introduction

Trichinellosis is a common food-borne parasitic zoonosis worldwide. Infection occurs by consuming raw or inadequately cooked meat containing muscle larvae of the *Trichinella* parasite. This parasite can infect a wide variety of hosts, including humans. *Trichinella* infection has been reported in 66 countries around the world. *Trichinella spiralis* is the major causative agent of trichinellosis. Outbreaks of trichinellosis in humans have been regularly reported worldwide, and serological analysis has indicated that more than 11 million people may be infected with *Trichinella* (Murrell and Pozio, 2011). Trichinellosis is regarded as an emerging or re-emerging infectious disease, particularly in developing countries, where cooking habits, poor sanitary conditions, and a lack of veterinary inspection facilitate infection (Gottstein et al., 2009). This zoonosis is both a public health hazard and an economic issue in terms of the porcine breeding industry and food safety (Bai et al., 2017). Therefore, in order to control and eradicate this disease, it is necessary to develop novel diagnostic and therapeutic methods for trichinellosis, which will require a comprehensive understanding of the parasite’s biology.

In recent years, the genome, transcriptome, epigenome, and proteome of *T. spiralis* have been reported and released in public databases, providing a greater understanding of the molecular basis of *T. spiralis* biology, host–parasite interactions, and pathogenesis (Mitreva et al., 2011; Gao et al., 2012; Liu et al., 2012, 2016). However, to the best of our knowledge, no post-translational modifications (PTMs), which occur during or after protein biosynthesis, have been described in *T. spiralis*, limiting further functional studies. PTMs regulate diverse protein properties, including folding, stability, localization, binding affinities, and activity, and they play important roles in a variety of cellular pathways and processes. Because of their substantial effects on protein functions and cellular processes, studies of protein PTMs have become more important in the post-genomic era (Walsh et al., 2005; Witze et al., 2007). To date, more than 461 different PTMs, such as phosphorylation, methylation, ubiquitination, malonylation, succinylation, glycosylation, and acetylation, have been identified to play a role in the functional regulation of many prokaryotic and eukaryotic proteins. These PTMs form a complex regulatory system for the dynamic control of cellular processes under various conditions (Yang and Seto, 2008).

Among these PTMs, lysine acetylation (Kac) is a highly dynamic and regulated modification that is defined as the reversible transfer of an acetyl group from the acetyl donor acetyl-CoA to the 𝜀-amino group of a lysine residue of a protein molecule. The Kac process is regulated by lysine acetyltransferases and deacetylases (KDACs), resulting in a regulatory range that is extensive and comparable with those of other major PTMs (Choudhary et al., 2009; Drazic et al., 2016). KDACs have been identified as drug target to develop novel anti-parasite treatments, and some selective KDAC ligands showed the potential for the treatment of parasitic diseases (Wang et al., 2015).

Recent studies have indicated that non-enzymatic chemical acetylation occurs broadly in eukaryotic cells, suggesting that this PTM may have various roles in protein function and cellular processes (Wagner and Hirschey, 2014; Olia et al., 2015). Kac was first identified in eukaryotic histones, where it is involved in regulating chromatin structure and transcription. In addition to its role in histones, Kac were found in a number of non-nuclear proteins which are located in almost every compartment of the cell (Friedmann and Marmorstein, 2013; Drazic et al., 2016). Protein acetylation outside the nucleus participates in diverse cellular physiological processes, including cell-cycle regulation, cytoskeletal dynamics, apoptosis, autophagy, and metabolism (Kim et al., 2006; Close et al., 2010; Wang et al., 2010; Zhao et al., 2010; Sun et al., 2015). In addition, acetylation has also been implicated to influence the persistence, virulence and antibiotic resistance of pathogenic bacteria (Xie et al., 2015).

Recent advances in antibody-based affinity purification of acetylated peptides and highly sensitive mass spectrometry (MS) have made significant contributions to the study of Kac at the whole-proteome level. Global Kac has been characterized in a number of eukaryotic and prokaryotic organisms (Jeffers and Sullivan, 2012; Zhang et al., 2013; Pan et al., 2014; Nie et al., 2015; Xie et al., 2015). These proteome-wide analyses of Kac have mapped large datasets of lysine-acetylated proteins and have confirmed that Kac is involved in various cellular events, especially in modulating metabolic processes. Based on these studies, it is proposed that the functional roles of acetylation in these processes are evolutionarily conserved in both eukaryotes and prokaryotes, and acetylation may thus rival phosphorylation.

Although Kac has been characterized in many organisms, the study of the lysine acetylome in helminths has been relatively limited, with only one nematode species (*Schistosoma japonicum*) being previously characterized (Hong et al., 2016). Given the general use of acetylation as a regulatory mechanism in different organisms, it can be speculated that *T. spiralis* also employs acetylation as a modification to modulate various aspects of its biology. Lending evidence to this hypothesis, *T. spiralis* appears to contain a great number of acetyltransferases and deacetylases in its genome (Mitreva et al., 2011).

To bridge this knowledge gap, in this study, proteomics was used in combination with immunoprecipitation in the first large-scale analysis of lysine-acetylated proteins in *T. spiralis*, identifying 3872 Kac sites in 1592 proteins. These lysine-acetylated proteins are involved in a variety of biological functions and are localized to multiple cellular compartments. Bioinformatics analysis showed that acetylation in *T. spiralis* modulates a wide range of cell processes. This study provides a global picture of the *T. spiralis* acetylome, which not only greatly expands our knowledge of helminth protein acetylation but also provides important information for the design of new effective drugs or vaccines for controlling trichinellosis.

## Materials and Methods

### Parasite Collection and Protein Extraction

Animal experiments were performed according to the guidelines of the National Institute of Health (publication No. 85–23, revised 1996) and Animal Experimentation Guidelines of Jilin University and were approved by the Animal Experimental Ethics Committee of Jilin University, China. *T. spiralis* (ISS534) parasites were maintained by serial passage in female BALB/c mice. *T. spiralis* muscle larvae (ML) were recovered from infected BALB/c mice at 35 days post-infection (dpi) using a standard HCl-pepsin digestion method. ML were washed manually in phosphate-buffered saline (PBS) at 37°C to remove any residual host proteins and were then collected for further lysine acetylome analysis.

Total proteins were extracted as previously described (Zhou et al., 2016) with minor modifications. In brief, parasites were lysed with lysis buffer containing 8 M urea, 10 mM dithiothreitol (DTT) and 0.1% protease inhibitor cocktail, followed by sonication. Cellular debris was then removed by centrifugation at 20,000 × *g* and 4°C for 10 min. Proteins contained in the supernatant were precipitated with cold 15% trichloroacetic acid (TCA) for 2 h at -20°C. After centrifugation, the pellet was washed three times with cold acetone, followed by dissolution in buffer containing 8 M urea and 100 mM NH_4_CO_3_ (pH 8.0). Protein concentration was measured using a 2-D Quant kit (GE Healthcare) according to the manufacturer’s instructions.

### Trypsin Digestion and High-Performance Liquid Chromatography (HPLC) Fractionation

Proteins were reduced with 10 mM DTT, alkylated with 20 mM iodoacetamide (IAA), and digested with trypsin (Promega) overnight at a 1:50 trypsin:protein mass ratio. To ensure complete digestion, additional trypsin was added at a 1:100 trypsin:protein mass ratio for 4 h for a second digestion. Tryptic peptides were separated into six fractions using high-pH reverse-phase HPLC, as previously described (Zhou et al., 2016). Separated peptides were then dried completely in a SpeedVac (Thermo Scientific) and stored at -80°C for further enrichment of acetylated peptides.

### Affinity Enrichment and Liquid Chromatography Electrospray Ionization Tandem Mass Spectrometry (LC-ESI–MS/MS) Analysis

Fractionated peptides were dissolved in NETN buffer containing 100 mM NaCl, 1 mM EDTA, 50 mM Tris–HCl, and 0.5% NP-40 (pH 8.0) and incubated with anti-acetyl lysine antibody beads (PTM Biolabs) at 4°C overnight with gentle shaking. After washing with NETN buffer and deionized H_2_O, the bound peptides were eluted with 0.1% trifluoroacetic acid, dried in a SpeedVac, and cleaned using C18 ZipTips (Millipore) according to the manufacturer’s instructions.

The obtained peptides were then analyzed by LC-ESI–MS/MS on a Q Exactive^TM^ Plus hybrid quadrupole-Orbitrap mass spectrometer (Thermo Fisher Scientific) coupled online to the EASY-nLC 1000 ultra-performance liquid chromatography (UPLC) system. The resulting MS/MS data were processed using MaxQuant with an integrated Andromeda search engine (v.1.5). Tandem mass spectra were queried against the UniProt *T. spiralis* database using the following parameters (Zhou et al., 2016): trypsin/P was specified as the cleavage enzyme and allowed up to four missed cleavages, five modifications per peptide, and five charges. Mass error was set to 10 ppm for precursor ions and 0.02 Da for fragment ions. Carbamidomethylation on Cys was specified as a fixed modification, and oxidation on Met, acetylation on Lys, and acetylation on the protein N-terminus were specified as variable modifications. False discovery rate (FDR) thresholds for the protein, peptide, and modification sites were set at 1%. The minimum peptide length was set at 7. The site localization probability was set as > 0.75.

### Bioinformatics Analysis

Gene Ontology (GO) annotation of the proteome was derived from the UniProt-GOA database^1^ according to Zhou et al. (2016). All identified acetylated proteins were classified using three categories of GO annotations: biological process, cellular component, and molecular function. The subcellular localization predication program, Wolfpsort (Horton et al., 2007), was used to analyze cellular localization data, and the InterPro database^2^ and InterProScan were used to compile functional descriptions of protein domains. Protein pathways were annotated using the Kyoto Encyclopedia of Genes and Genomes (KEGG) (Kanehisa et al., 2004). Amino acid sequence motifs in identified protein were analyzed using Soft motif-x, and a position-specific heat map was generated using WebLogo 3.4^3^. Secondary structures of all acetylated proteins were predicted using NetSurfP software. Fisher’s exact test was used to analyze GO and KEGG pathway and domain enrichment. Terms with adjusted *p*-values < 0.05 were considered significantly enriched. The Search Tool for Retrieval of Interacting Genes/Proteins (STRING) database^4^ was used for analysis of the protein–protein interaction (PPI) network. The interaction network form STRING was visualized using Cytoscape (version 3.0) software (Bauer-Mehren, 2013).

## Results and Discussion

### Identification and Analysis of Lysine-Acetylated Proteins in *T. spiralis*

**FIGURE 1:**
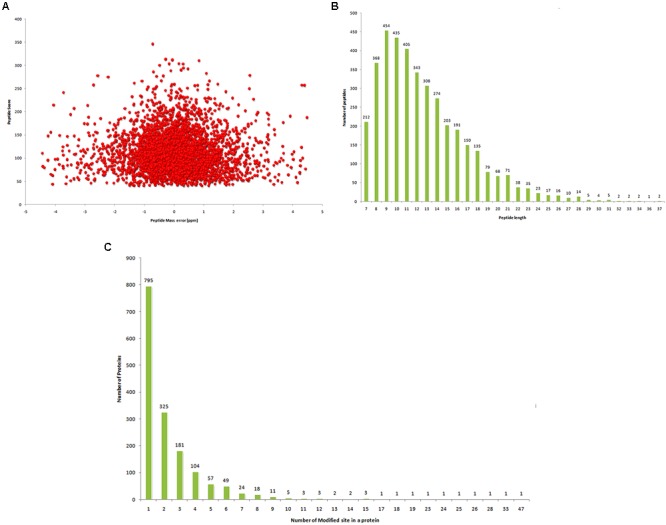
Proteome-wide identification of lysine acetylation sites in *T. spiralis.* **(A)** Mass error distribution of all identified peptides. **(B)** Distribution of acetylated peptides based on length. **(C)** Distribution of acetylated proteins based on number of acetylation sites.

Sequencing of the *T. spiralis* genome revealed the existence of a number of acetyltransferase and deacetylase orthologs, suggesting that Kac may play a vital role in *T. spiralis* development and metabolism (Mitreva et al., 2011). To comprehensively characterize protein acetylation in *T. spiralis*, analysis of Kac was carried out at the proteome level using tryptic digestion, anti-acetyl lysine antibody enrichment, and high-resolution LC-MS/MS. Generally, the distribution of mass errors was near zero, and most were ≤ 10 ppm (**Figure 1A**). Moreover, all lysine-acetylated peptides were between 7 and 37 amino acids in length, which is consistent with tryptic peptides (**Figure 1B**). These results indicated that the sample preparation method was adequate and that the modified peptide data obtained from MS was highly accurate.

In total, 3872 Kac sites distributed across 1592 acetylated proteins were identified (Supplementary Table S1). This exceeds the level of Kac that has so far been reported in *S. japonicum* (Hong et al., 2016), indicating the essential role of Kac in *T. spiralis*. The acetylated proteins of *T. spiralis* contained various numbers of acetylation sites, ranging from 1 to 47 in the putative myosin head protein (E5SJK9) (**Figure 1C**), with an average frequency of 2.4 sites per protein. Among all acetylated proteins, approximately half (795/1592) contained only one acetylated site, and the percentages of proteins with two, three, four, or > 4 modification sites were 20.4, 11.4, 6.5, and 11.7%, respectively. Acetylation of non-histone proteins at multiple sites have shown to modulate every step of cellular processes from signaling to transcription to protein degradation through various molecular mechanisms (Huang and Berger, 2008; Spange et al., 2009). The role of multiple acetylation sites in regulating protein function remains to be elucidated.

### Analysis of Kac Motifs

**FIGURE 2:**
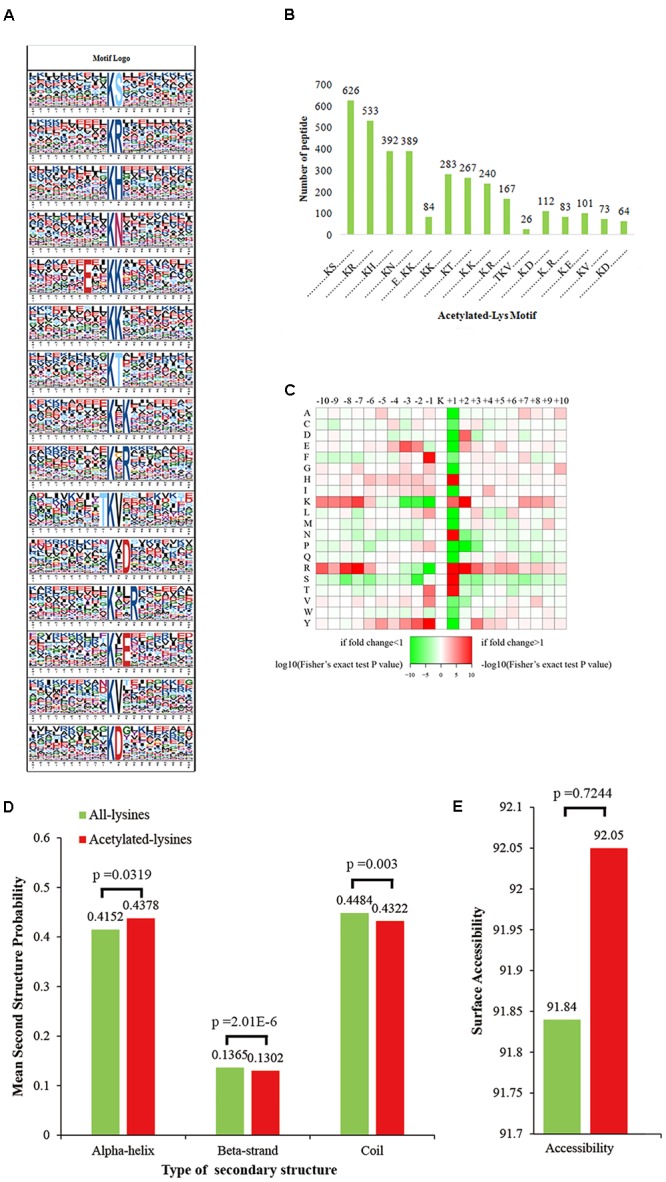
Bioinformatics study of lysine-acetylated sites. **(A)** Probable sequence motifs of *T. spiralis* acetylation sites consisting of 20 residues surrounding the targeted lysine residue, identified using Motif-X. **(B)** Number of identified peptides containing acetylated lysines and each motif. **(C)** Heat map showing the relative frequencies of amino acids in specific positions, including enrichment (red) or depletion (green) of amino acids flanking the acetylated lysine in *T. spiralis* proteins. **(D)** Probabilities of lysine acetylation in different protein secondary structures (alpha-helix, beta-strand, and coil). **(E)** Predicted surface accessibility of acetylation sites. All lysine sites are shown in green, and acetylated lysine sites are in red.

Previous studies in both eukaryotic and prokaryotic cells have shown preferences for amino acid residues at specific positions surrounding acetylated lysines (Nie et al., 2015; Zhou et al., 2016). In our study, a total of 15 conserved amino acid sequence motifs from -10 to +10 surrounding the acetylated lysine were defined from among 3440 peptides (accounting for 88.8% of the total identified peptides). These motifs included KacS, KacR, KacH, KacN, E^∗∗^KacK, KacK, KacT, K^∗^Kac, Kac^∗^R, TKacV, Kac^∗^D, Kac^∗∗^R, Kac^∗^E, KacV, and KacD, an asterisk indicates a random amino acid residue. These motifs exhibited different abundances (**Figure 2A** and Supplementary Table S2, with the KacS, KacR, KacH, and KacN motifs being the most frequent and accounting for 18.7, 16.1, 11.4, and 11.3% of all identified peptides, respectively (**Figure 2B**). In addition, most of the conserved residues were located at the ± 1 or ± 2 positions of the Kac sites. The motif analysis results suggest that two particular types of residues are found near Kac sites. Enrichment of residues with major hydrophilic side chain groups, such as serine (S), arginine (R), and aspartic acid (D), was observed at the +1 position, and enrichment of threonine (T) with hydrophobic side chain groups was observed at the -1 position. These results suggest that amino acid residues with hydrophilic and hydrophobic side chains may be more easily attacked by Kac.

Studies of acetylation motifs have shown that aromatic amino acids frequently surround modification sites (Hong et al., 2016; Wang et al., 2017). Unexpectedly, we noticed only one aromatic amino acid (histidine, H) surrounding acetylated lysines, suggesting that the lysine acetyltransferases of *T. spiralis* might not prefer polypeptides with aromatic amino acid residues as substrates. Furthermore, some acetylated lysine motifs found in *T. spiralis* have also been identified in other organisms, such as KacH in humans (Shao et al., 2012) and *Mycobacterium tuberculosis* (Xie et al., 2015); Kac^∗∗^R in humans (Shao et al., 2012); Kac^∗^E in rats (Lundby et al., 2012); and KacR, KacT, and KacV in *Toxoplasma gondii* (Jeffers and Sullivan, 2012), confirming that Kac is a highly conserved modification among various species. Moreover, LceLogo heat maps of the amino acid compositions surrounding the acetylation sites showed that the frequencies of serine (S), threonine (T), and histidine (H) in positions -2 to + 2 were the highest, while the frequency of tryptophan (W) was the lowest (**Figure 2C**) in regions surrounding these sites. Based on these findings, we infer that proteins with S, T, and H but not W around lysine residues are the favored targets of lysine acetyltransferases in *T. spiralis*. A structural analysis of the acetylated proteins was carried out using NetSurfP software in order to understand the relationship between acetylation and the presence of specific protein secondary structures in *T. spiralis*. As shown in **Figure 2D**, acetylation sites (56.8%) were more common in regions with ordered secondary structures.

Among the acetylated sites, 43.8% of the acetylated sites were located in alpha-helices, and 13.2% were located in beta-strands. The remaining 43.2% of the acetylated sites were distributed in unstructured protein regions. These results suggest that acetylases may prefer ordered structural regions to disordered regions in *T. spiralis* proteins, which differs from findings of the acetyl proteomes of humans and *S. japonicum* (Hong et al., 2016). The acetylation sites in these species have been found to be located primarily in disordered protein regions, with less acetylation in ordered protein structures, suggesting that secondary structure preferences at lysine-acetylated sites may vary among species. However, a similar distribution pattern of secondary structures was observed for non-acetylated lysines in the proteome of *T. spiralis*, indicating that there is no tendency toward acetylation in *T. spiralis*. Finally, the surface accessibility of the acetylated lysine sites was also studied. The results showed that the exposure of acetylated residues on the protein surface is very similar to that of non-acetylated lysine residues (**Figure 2E**). Therefore, Kac may not be affected by the surface properties of proteins in *T. spiralis.*

### Evolutionary Conservation of Kac in *T. spiralis*

**FIGURE 3:**
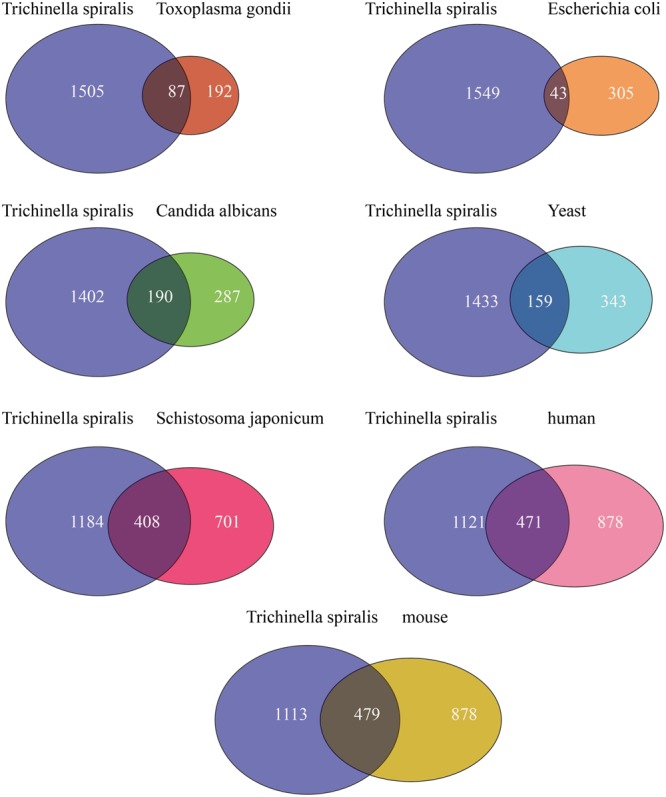
Analysis comparing the *T. spiralis* acetyl proteome with those of *E. coli*, budding yeast (*S. cerevisiae*), *C. albicans, T. gondii, S. japonicum*, mouse, and human. Venn diagram describing the relationships among lysine-acetylated proteins from these species.

Increasing evidence has indicated that Kac maybe evolutionarily conserved among various species (Zhou et al., 2016). To further improve our understanding of the evolution of acetylation in *T. spiralis*, we compared the acetylated proteins in *T. spiralis* with sacetylated proteins that have been reported in other organisms based on the quantity of orthologs. The published acetylated proteins of *Escherichia coli* (Zhang et al., 2013), budding yeast (*Saccharomyces cerevisiae*) (Henriksen et al., 2012), *Candida albicans* (Zhou et al., 2016), *T. gondii* (Jeffers and Sullivan, 2012), *S. japonicum* (Hong et al., 2016), mouse (liver tissue), and human [cervical cancer (HeLa) cells] (Weinert et al., 2013) were selected. These acetylome data are comparable, as similar methods were used for the systematic identification of Kac in each of these species. Among the 1592 acetylated proteins in *T. spiralis*, 408 (35.5%) proteins had orthologs in the *S. japonicum* acetylome, 479 (35.2%) had orthologs in the mouse acetylome, 471 (36.3%) had orthologs in the human acetylome, 159 (31.6%) had orthologs in the *S. cerevisiae* acetylome, 190 (39.8%) had orthologs in the *C. albicans* acetylome, 87 (30%) had orthologs in the *T. gondii* acetylome, and 43 (12.3%) had orthologs in the *E. coli* acetylome (**Figure 3** and Supplementary Table S3). The lowest ortholog rate was between *T. spiralis* and *E. coli*, and this could reflect differences in cellular structures between prokaryotes and eukaryotes. The highest overlap was between *T. spiralis* and *C. albicans*, which can be explained by the genetic relatedness between them and is likely due to a similar subcellular distribution of acetylated proteins. These results indicate that acetylation occurs frequently in both eukaryotes and prokaryotes with certain species.

### Functional Annotation and Cellular Localization of Acetylated Proteins in *T. spiralis*

**FIGURE 4:**
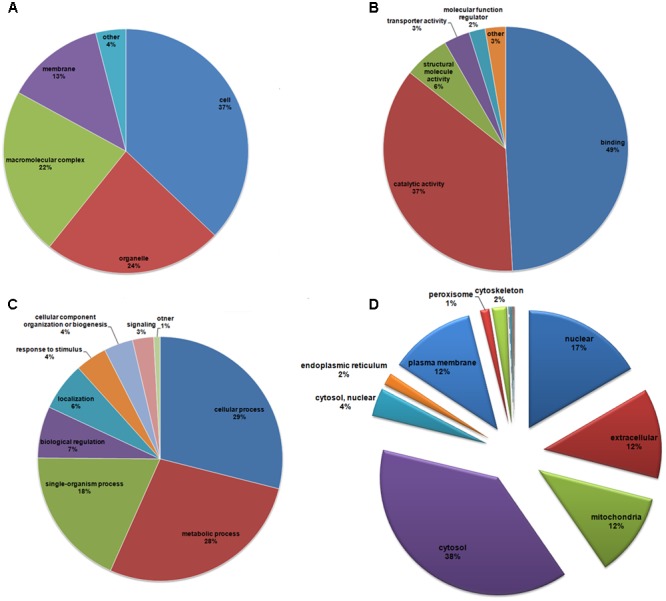
Gene Ontology functional classification and subcellular location information of identified acetylated proteins. **(A)** Acetylated proteins classified according to cellular components. **(B)** Acetylated proteins classified according to biological process. **(C)** Acetylated proteins classified according to molecular function. **(D)** Subcellular localizations of identified acetylated proteins.

To functionally characterize the identified lysine-acetylated proteins, we first investigated the GO functional classifications of all acetylated proteins (**Figure 4** and Supplementary Table S4). As shown in **Figure 4A**, acetylated proteins were distributed among a variety of cellular compartments, primarily in cells (37%), organelles (24%), macromolecular complexes (22%), and membranes (13%). According to the molecular function classification, the largest group of acetylated proteins was composed of those involved in the binding of various targets, accounting for 40% of all identified acetylated proteins (**Figure 4B**). This result suggests that acetylation may play essential roles in regulating PPIs in *T. spiralis*. The second largest group in the molecular function category included proteins related to catalytic activity (37%). Enzymes play important roles in regulating biochemical reactions in organisms because of their catalytic activity. Studies have indicated that reversible Kac can regulate metabolic enzymes by increasing or decreasing enzyme quantity, catalytic activity, or substrate accessibility (Xiong and Guan, 2012). Our results are consistent with those of previous studies, in which a considerable number of lysine-acetylated proteins with catalytic activity were enzymes involved in metabolic processes (Jeffers and Sullivan, 2012; Hong et al., 2016).

In the biological process classification of the GO annotation of acetylated proteins, two main classes of acetylated proteins were involved in cellular and metabolic processes, accounting for 29 and 28% of identified proteins, respectively (**Figure 4C**). Previous studies have demonstrated that reversible Kac is an emerging regulatory mechanism for proteins involved in metabolism and cellular processes in bacteria (Pan et al., 2014), parasites (Jeffers and Sullivan, 2012), animals, and humans (Weinert et al., 2013). Here, our results indicate that Kac may also play a critical role in *T. spiralis* metabolism and cellular regulation.

The localization of proteins to various subcellular compartments is important for allowing proteins to perform their necessary functions. To obtain a profile of acetylated proteins throughout the cell, we analyzed the subcellular localization of the 1592 acetylated proteins in *T. spiralis* (**Figure 4D**). Analysis of the acetylated proteins showed that the majority of identified Kac proteins were localized to the cytoplasm (38%), followed by the nucleus (17%), and mitochondria (12%). This result is consistent with those of the biological process analysis, which showed that most acetylated proteins were involved in protein cellular metabolism and protein synthesis. Importantly, we also found that a number of acetylated proteins were cytoplasmic membrane (12%) or extracellular (12%) proteins, which could function in regulating cuticular layer-related proteins, impacting the interactions between *T. spiralis* and host cells. In addition, 1% of the acetylated proteins in this study were located in the peroxisome, where fatty acids are activated and converted into acyl-CoA before being transformed into sucrose.

### Functional Enrichment Analysis

**FIGURE 5:**
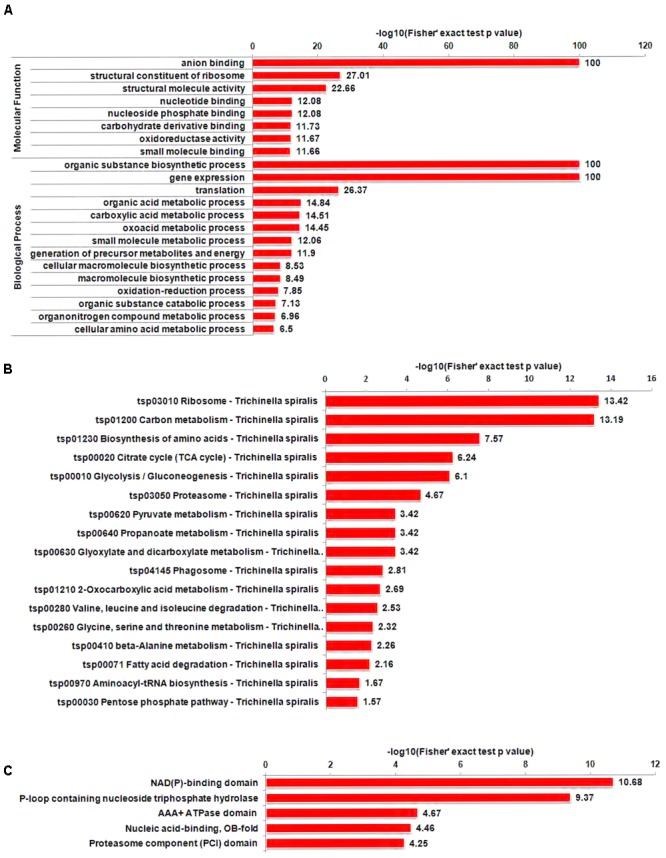
Enrichment analysis of acetylated proteins in *T. spiralis*. **(A)** GO-based enrichment analysis of acetylated proteins in terms of molecular function, cellular component, and biological process. **(B)** KEGG pathway enrichment analysis. **(C)** Protein domain enrichment analysis.

In order to reveal the preferred targets of Kac, we performed a GO enrichment analysis of the acetylation data. The GO enrichment analysis of the molecular function category further demonstrated that acetylated proteins were significantly enriched in those related to binding activity and the structural constituents of ribosomes (**Figure 5A**). GO enrichment analysis based on the biological process category showed that acetylated proteins were enriched in those involved in biosynthetic and metabolic processes (**Figure 5A**), suggesting that these processes may be precisely regulated by protein acetylation. As acetylated proteins may be involved in both of these fundamental processes, Kac appears to be a pivotal regulatory mechanism in *T. spiralis*.

To gain in-depth insights into the functions of Kac proteins in *T. spiralis*, KEGG enrichment analysis was also carried out (**Figure 5B**). Our data showed that the acetyl proteome of *T. spiralis* was highly enriched in proteins involved in protein biosynthesis and the metabolism of three types of biological macromolecules (**Figure 5B**). The significant enrichment of proteins involved in various pathways, including translation, transcription, and metabolism, among acetylated proteins has also been reported in other eukaryotes and prokaryotes (Nie et al., 2015; Hong et al., 2016; Zhou et al., 2016), confirming the essential role of Kac in various organisms.

Small protein motifs involved in protein interactions are also regulated by PTMs. Thus, we performed a domain enrichment analysis. In total, acetylated proteins were significantly enriched for 25 protein domains, including the NAD(P)-binding domain, AAA+ ATPase domain, and nucleic acid-binding (OB-fold) domain (**Figure 5C**). Proteins with these functional domains are involved in a variety of metabolic pathways. A previous study indicated that enzymes with an NAD(P)-binding domain catalyze redox reactions, suggesting that Kac plays an important role in the stress response (Hua et al., 2014). The OB-fold is a compact structural motif that recognizes single-stranded and unusually structured nucleic acids, and it plays a critical role in DNA replication, DNA repair, transcription, translation, and telomere maintenance (Theobald et al., 2003), making these proteins attractive anti-parasitic drug targets. In our study, a large number of proteins containing nucleic acid-binding domains, such as helicase, tyrosine–tRNA ligase, 40S ribosomal protein S11, GTP-binding protein, asparaginyl–tRNA synthetase, and nuclease domain-containing protein 1, were found to have different numbers of acetylation sites. Prevalent acetylation of proteins containing nucleic acid-binding domains may play an important role in directing the recognition of single-stranded and unusually structured nucleic acids. Overall, our results indicate that acetylated proteins are widely distributed throughout the cell, with important effects on a variety of processes in *T. spiralis*.

### PPI Analysis

Protein–protein interactions occur widely in all cell types and are important in regulating cellular processes. To better understand how interactions among acetylated proteins may affect the regulation of *T. spiralis* physiology and development, we assembled a PPI network containing all of the identified acetylated proteins using Cytoscape. The results showed that 666 acetylated proteins represented network nodes that were connected by 9446 direct physical interactions, with a combined score > 0.90 obtained from the STRING database. The majority of proteins in this interaction network contain multiple Kac sites, and the network suggests that the physiological interactions among these acetylated proteins are involved in multiple protein interaction networks and control various signaling pathways in *T. spiralis*. A complete *T. spiralis* network of acetylated proteins is shown in Supplementary Table S5 and represents the first high-quantity interaction network of acetylated proteins in a foodborne parasite.

On the basis of the interaction network, we retrieved four highly interconnected interaction clusters of acetylated proteins using the MCODE algorithm in Cytoscape.

**FIGURE 6:**
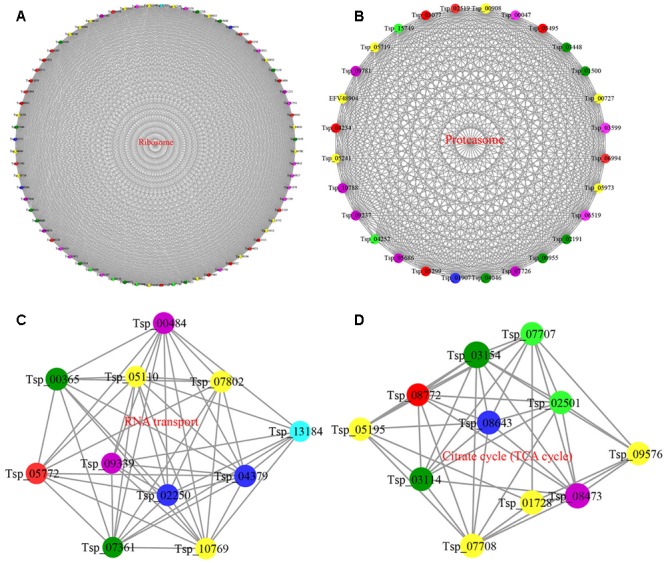
Interaction networks of acetylated proteins in *T. spiralis*. **(A)** Interaction network of acetylated proteins associated with the ribosome. **(B)** Interaction network of acetylated proteins associated with the proteasome. **(C)** Interaction network of acetylated proteins associated with RNA transport. **(D)** Interaction network of acetylated proteins associated with the TCA cycle.

The top cluster, with 73 acetylated proteins (cluster I), consisted of ribosome-associated proteins that were connected in a PPI network with a relatively high density (**Figure 6A**). Clusters II and III consisted of proteins associated with the proteasome and RNA transport, whereas the fourth highest-ranking complex consisted of proteins involved in the tricarboxylic acid (TCA) cycle (**Figures 6B–D**). These subnetworks also have relatively high densities, and many of the acetylated proteins in the network contain multiple Kac sites. These findings suggest that the protein metabolic metabolite machinery is probably regulated by Kac in *T. spiralis*; the identified proteins were also involved in central metabolic pathways.

### Analysis of Histone Kac

Histone acetylation occurs in diverse organisms and is now known to play a critical role in chromatin remodeling, DNA repair, and the epigenetic regulation of gene expression (Campos and Reinberg, 2009). The acetylation of histones 3 and 4 is commonly associated with active transcription, and histone acetylation at H3K27 play critical roles in regulating enhancer activities in cooperation with histone methylation (Hyun et al., 2017; Shen et al., 2017; Youn, 2017) Histone acetylation is also essential for parasite survival. In *S. mansoni*, histone deacetylase inhibitors induce mortality and apoptosis, which are associated with the increased expression of the caspase 3 and 7 transcripts due to histone H4 hyperacetylation. Thus, the histone deacetylase of *S. mansoni* may be an important target for schistosomiasis chemotherapy (Dubois et al., 2009). Many specific histone acetylation sites have been characterized in various parasites. The acetylation of H4K8 and H4K16 in *Giardia lamblia* plays a crucial role in regulating encystation, which protects the parasite from harsh environmental conditions (Carranza et al., 2016). Similarly, variations in the levels of H4K8 acetylation are important in regulating stage-specific gene expression throughout the *Plasmodium falciparum* life cycle (Carranza et al., 2016). In the present study, we identified six histone proteins with multiple Kac sites (Supplementary Table S6), with H2B (E5RY73) being heavily acetylated at eight lysine residues. Some acetylated lysine sites identified in our study have also been found in other parasites, such as *T. gondii* (Jeffers and Sullivan, 2012) and *P. falciparum* (Miao et al., 2013). However, further research is required to determine the significance of these modifications in *T. spiralis* and their roles in development and infection.

### Protein Acetylation Regulates Diverse Metabolic Pathways in *T. spiralis*

Recently, proteomic studies into acetylation have indicated that abundant proteins are acetylated in various organisms, suggesting that acetylation is a common mechanism of metabolic regulation (Hong et al., 2016; Zhou et al., 2016). Consistent with previous results, our study showed that 138 acetylated proteins were involved in multiple metabolic pathways. Here, we focused on two of these pathways: glycometabolism and protein metabolism.

#### Glycometabolism

**FIGURE 7:**
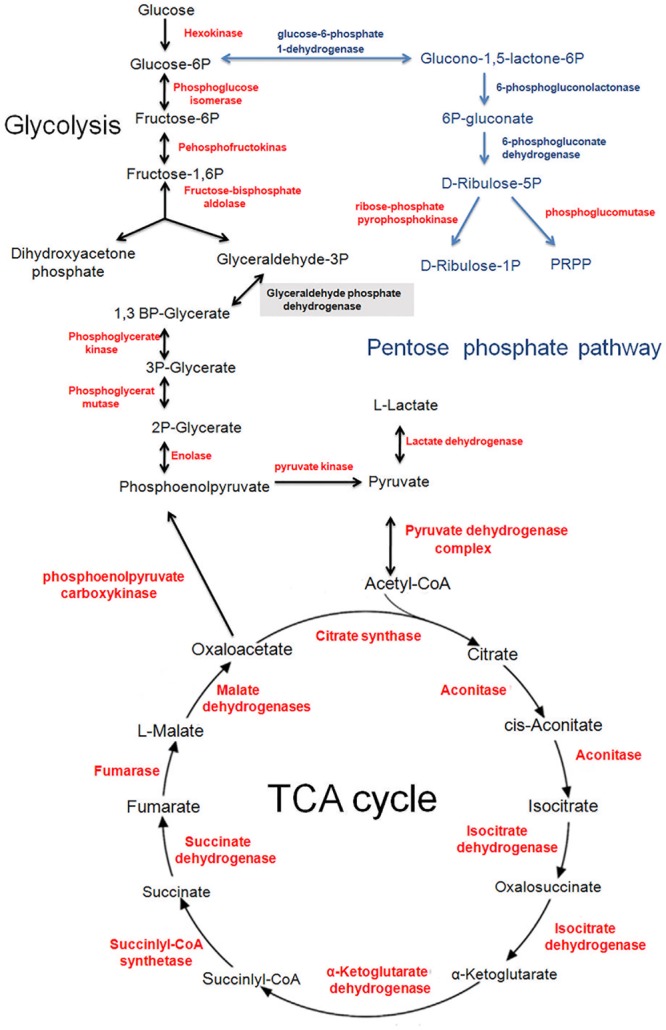
Lysine-acetylated proteins involved in the central carbon metabolic network in *T. spiralis*. Identified acetylated enzymes are shown in red.

The acetylation of proteins involved in glycometabolism (mainly enzymes) has been showed to play a crucial regulatory role in processes such as parasite development and growth (Miao et al., 2013; Hong et al., 2016). In our study, a large number of enzymes in these pathways were observed to undergo acetylation in *T. spiralis* (**Figure 7**), indicating that acetylation also participates in regulating glycometabolism in *T. spiralis.*

*Trichinella spiralis* larvae ferment glycogen under aerobic or anaerobic conditions via phosphorylative glycolysis, converting it to a mixture of volatile fatty acids (Agosin and Aravena, 1959). We found that almost all glycolytic enzymes involved in the conversion of glucose to pyruvate were substrates of Kac (15 proteins). Previous studies have found that seven out of ten glycolytic enzymes are acetylated in the *E. coli* acetylome (Zhang et al., 2013), while only glucose-6-phosphate isomerase is not acetylated in *P. falciparum* (Miao et al., 2013). Similarly, all enzymes involved in glycolysis are acetylated in the *S. japonicum* acetylome (Hong et al., 2016). In our study, only glyceraldehyde-phosphate dehydrogenase (GAPDH) was not found to be acetylated. In glycolysis, GAPDH acetylation increases glycolytic activity. Thus, the acetylation of GAPDH is important for controlling carbon flux, which is beneficial for glycolysis in cells supplied with glucose (Li et al., 2014). As the GAPDH protein of *T. spiralis* was not acetylated in our study, other enzymes, such as fructose-bisphosphate aldolase or phosphoglycerate kinase (PGK), with six and eight Kac sites, respectively, may be the main targets for the regulation of glycolysis in *T. sp*iralis. Previous study have indicated that acetylation of fructose 1,6-bisphosphate aldolase at K14 increased enzymatic activity (Kim et al., 2006). PGK is another central enzyme in the glycolysis pathway, catalyzing the conversion of 1,3-diphosphoglycerate to 3-phosphoglycerate and generating a molecule of ATP. The proliferation of cancer cells demands a lot of energy and is mainly dependent on anaerobic glycolysis for the conversion of glucose, a phenomenon known as the “Warburg effect.” Glycolysis is much less potent than mitochondrial oxidative phosphorylation in terms of ATP production, as only two enzymes, PGK and pyruvate kinase, control the production of ATP during anaerobic glycolysis in cancer cells. A recent study found that the acetylation of PGK at K323 enhances PGK activity and promotes cancer cell proliferation and tumorigenesis (Hu et al., 2017). In our study, the lysine sites in PGK were heavily acetylated, which may facilitate nutrient absorption and *T. spiralis* growth.

In addition, in our study, enolase exhibited the greatest number of acetylated sites (12 sites). In addition to their glycolytic function, enolases were recently shown to interact with plasminogen to induce fibrinolytic activity and to promote the migration of larval stages through tissues by plasmin-mediated proteolysis, such as the degradation of the host’s extracellular matrix. Enolase can induce some protection against schistosomal infection and also inhibits egg production in schistosomes (Yang et al., 2010). Therefore, enolase is considered a potential target for the development of new anti-parasitic vaccines and drugs. However, more work is required to fully elucidate the role of acetylation in controlling the function of enolase.

Another interesting pathway, the pentose phosphate pathway contained 10 acetylated proteins according to our results. This pathway provides reductive potential by producing NADPH, a cellular reductant that protects against antioxidant enzyme activity. It has also been shown to play an essential protective role against metalloid-mediated oxidative stress by stringently regulating the accumulation of reactive oxygen species (ROS) (Ghosh et al., 2017). Thus, the pentose phosphate pathway is considered to play an essential role in protecting cells against oxidative stress (Ghosh et al., 2015). Based on our results, we speculate that Kac plays a vital role in protecting the biomolecules of *T. spiralis* from oxidative damage.

Carbon is metabolized continuously in parasites and is essential for energy circulation and parasite survival. The TCA cycle is essential for supplying the energy needed for numerous cellular functions. Unlike *Schistosoma*, which completely relies upon glucose fermentation and the formation of lactate to meet its energy requirements, *T. spiralis* utilizes the oxidation of carbohydrates to CO_2_ and water via the TCA cycle as its major energy source (Goldberg, 1957).

A key entry point for carbon into the TCA cycle for energy production is the pyruvate dehydrogenase complex (PDC), which catalyzes the overall conversion of the glycolytic degradation products of carbohydrate metabolism and pyruvate to acetyl-CoA and CO_2_. The PDC is composed of three different subunits: pyruvate decarboxylase (E1, PDH), dihydrolipoamide transacetylase (E2, DLAT), and dihydrolipoamide dehydrogenase (E3, DLD). All of these subunits are required to form NADH from the oxidation of pyruvate. In our study, we identified five acetylated proteins in this complex. Of note, the PDC in glycolysis was acetylated at 22 sites among its three subunits, with six and nine lysine sites on DLAT and DLD, respectively, modified by Kac. In human cancer cells, K321 acetylation of PDHA1 and K202 acetylation of PDH phosphatase (PDP1) inhibit PDC activity (Fan et al., 2014). Thus, the intensive acetylation of acetyl-CoA metabolism-related enzymes in *T. spiralis* may regulate the catalytic activity of the complex, especially PDH, and control its functional balance in *T. spiralis*.

Many mitochondrial proteins that play an essential role in energy metabolism exhibit a high level of acetylation in many organisms (Hong et al., 2016; Zhou et al., 2016). Previous studies have shown that the acetylation of malate dehydrogenase 1 (MDH1), a key enzyme in the TCA cycle, significantly enhanced its enzymatic activity. This led to a subsequent increase in intracellular NADPH levels, promoting adipogenic differentiation in preadipocyte cells (Kim et al., 2012). We identified eight acetylation sites in malate dehydrogenase (MDH) in this study, including the acetylation of lysines 108, 119, 145, 168, 178, 228, 312, and 314. This protein was also found to be acetylated in *S. japonicum* (Hong et al., 2016). In our study, other enzymes involved in the TCA cycle were also identified as Kac targets, confirming that acetylation has a potential conserved function in the regulation of the TCA cycle in both eukaryotic and prokaryotic cells.

#### Protein Metabolism

In addition its regulation of glycometabolism, Kac was also observed in proteins regulating protein expression and proteolysis. In this study, we identified 120 lysine-acetylated proteins related to protein synthesis, accounting for 7.5% of the total lysine-acetylated proteins (Supplementary Table S1). Our results are consistent with those of previous studies showing that ribosomal proteins and aminoacyl–tRNA synthetases are the main targets of acetylation in both prokaryotes and eukaryotes. In *E. coli*, 22% of the acetylated proteins are involved in the translational machinery or processes (Zhang et al., 2013), while this figure is 15% in *Toxoplasma* (Jeffers and Sullivan, 2012*)*. Among these proteins, elongation factor 1-α of *T. spiralis* was heavily acetylated at 15 lysine residues, similar to the acetylation of its orthologs in other eukaryotes (humans, rats, and *Toxoplasma*) (Jeffers and Sullivan, 2012; Lundby et al., 2012; Shao et al., 2012). tRNA synthetases represent potential drug targets for the prevention and control of parasitic infections. For example, inhibitors of isoleucine–tRNA synthetase and seryl–tRNA synthetase can suppress *P. falciparum* growth (Miao et al., 2013). In this study, 20 aminoacyl–tRNA synthetases were found to have acetylated lysine residues, reflecting a higher frequency of acetylation than among some other eukaryotes.

The ubiquitin–proteasome system provides a major mechanism for regulating the degradation of cell-cycle regulators and potentially toxic misfolded proteins, and it is required for maintaining cellular protein homeostasis in eukaryotic cells. The core of this large protease is the 20S proteasome, a barrel-shaped structure made of a stack of four heptameric rings. Kac has dual roles in regulating protein degradation in the proteasome. A direct role of protein acetylation involves competing with protein ubiquitination for lysine sites, preventing the proteasomal degradation of target proteins. In addition, in some cases, the acetylation of a specific lysine can unexpectedly create a high-affinity binding site for other proteins, leading to its subsequent ubiquitination and degradation (Caron et al., 2005). In our study, the 20S core particle of the proteasome was frequently modified by Kac (eight proteins), particularly the α subunits (Supplementary Figure S1). Accordingly, three types of amino acid degradation-related enzymes associated with the degradation of valine, leucine, and isoleucine were acetylated (Supplementary Table S1), suggesting that acetylation plays a direct regulatory role in the ubiquitin–proteasome system and influences proteolytic processes in *T. spiralis*.

Phagocytosis, a multistep process that is completely dependent on cytoskeletal complexes (Radulovic and Godovac-Zimmermann, 2011; Drazic et al., 2016), has been shown to play crucial roles in the acquisition of nutrients, cell proliferation, and virulence in parasites (Mansuri et al., 2014). After binding to a cell surface receptor, the actin cytoskeleton is locally remodeled to provide the necessary force for the formation of phagocytic cups and phagosomes. The spatial and temporal regulation of actin dynamics is required for controlling the various stages of phagocytosis, and interference with actin dynamics reduces phagocytosis (Mansuri et al., 2014). Kac have been suggested to play a crucial role in maintaining actin structure and dynamics and facilitating stress fiber formation and actomyosin interactions (Choudhary et al., 2009). Another group of important cytoskeleton-associated proteins are microtubules, which are formed from α- and β-tubulin heterodimers. The acetylation of α-tubulin has been shown to affect the kinetics of tubulin polymerization and the localization of the motor protein kinesin. The hyperacetylation of microtubules causes the misregulation of transport by kinesin motors (Yang and Seto, 2008; Friedmann and Marmorstein, 2013). In addition, investigations into the antitumor efficacies of histone acetyltransferase inhibitors have shown that the acetylation level of tubulin may be a predictive biomarker of its sensitivity to Histone acetyltransferase inhibition (Di Martile et al., 2016). In our study, the cytoskeleton-associated proteins actin and tubulin, which are also involved in phagocytosis, were found to be acetylated (Supplementary Figure S2), indicating that the acetylation of cytoskeleton-associated proteins may participate in the regulation of phagocytosis in *T. spiralis*.

Recently, it has become evident that two major endoplasmic reticulum (ER) proteins, calnexin and calreticulin, are crucial regulators of particle uptake into phagosomes, controlling the opening and closing of the cups in *Dictyostelium discoideum* (Muller-Taubenberger et al., 2001). During phagocytosis, an acidic phagosomal environment generated by V-ATPases is of pivotal importance for the degradation of macromolecules. Unexpectedly, we found that these proteins important in phagocytosis were heavily acetylated in our study. Among these, eight ATPases were found to be acetylated at multiple sites (Supplementary Figure S2). To the best of our knowledge, this is the first time that abundant proteins involved in phagocytosis have been found to be acetylated in an organism, confirming that Kac is of great significance in the regulation of phagocytosis.

The complete life-cycle of *T. spiralis* occurs in a single host and can be divided into three main stages: ML, adult worms, and newborn larvae (NBL). These occupy two distinct intracellular niches: the intestinal epithelium and skeletal muscle cells. Among these developmental stages, *T. spiralis* has developed fascinating strategies for adapting to different environments and successfully establishing infection, such as the switching of parasite surface antigens. Studies have indicated that acetylation can regulate antigenic variation and developmental transitions in parasites, and these are principally determined by strong selective pressures (Coleman et al., 2014; Carranza et al., 2016). Thus, it would be of great interest to investigate the global acetyl-proteome of *T. spiralis* in other developmental stages and compare the levels of acetylation of the proteomes. This will facilitate the dissection of the role of the lysine acetylome in regulating the developmental transitions of *T. spiralis* and its establishment of infection under the selective pressures of different living environments.

## Conclusion

We have produced the first large-scale acetyl-proteome of *T. spiralis*, an important foodborne parasite, showing abundant Kac among *T. spiralis* proteins. The lysine acetylome in the present study provides a good foundation for in-depth studies of the functions of reversible Kac in the growth and development of *T. spiralis* and other nematodes. Identifying the functions of the acetylation of target proteins may also help to design specific and effective drugs or vaccines to prevent and control trichinellosis.

## Author Contributions

ML and YZ designed the research. YY, MT, XB, XiL, and IV carried out the data acquisition, analysis, and interpretation. PZ and WC performed the *T. spiralis* infection experiments. YY, MT, and XB contributed to the writing of the manuscript. XC and XuL contributed to improvement of the manuscript. All authors contributed to the study concept and critical revision of the manuscript for important intellectual content.

## Conflict of Interest Statement

The authors declare that the research was conducted in the absence of any commercial or financial relationships that could be construed as a potential conflict of interest.

## Footnotes

1 http://www.ebi.ac.uk/GOA/

2 http://www.ebi.ac.uk/interpro/

3 http://weblogo.threeplusone.com/create.cgi

4 http://string-db.org/
