# Supplementary material for: Comprehensive Proteomic Analysis of Lysine Acetylation in the Foodborne Pathogen Trichinella spiralis
Source: Front Microbiol. 2018 Jan 11;8:2674. doi: 10.3389/fmicb.2017.02674 (PMC5768625; doi:10.3389/fmicb.2017.02674)
Supplement: Supplementary file 6 [file Table_6.docx]

Table S6 Acetylation sites and acetylated histones of identified in this Study

| Protein accession | Position | Amino acid | Protein names | Gene names |
| --- | --- | --- | --- | --- |
| **H2A** | | | | |
| E5RY74 | 6 | K | Histone H2A | Tsp_07128 |
| E5RY74 | 9 | K | Histone H2A | Tsp_07128 |
| E5RY74 | 11 | K | Histone H2A | Tsp_07128 |
| **H2B** | | | | |
| E5S7K8 | 4 | K | Histone H2B | H2B-VII |
| E5S7K8 | 8 | K | Histone H2B | H2B-VII |
| E5S7K8 | 11 | K | Histone H2B | H2B-VII |
| E5S7K8 | 12 | K | Histone H2B | H2B-VII |
| E5S7K8 | 15 | K | Histone H2B | H2B-VII |
| E5S7K8 | 18 | K | Histone H2B | H2B-VII |
| E5S7K8 | 21 | K | Histone H2B | H2B-VII |
| E5RY73 | 4 | K | Histone H2B | Tsp_07127 |
| E5RY73 | 8 | K | Histone H2B | Tsp_07127 |
| E5RY73 | 11 | K | Histone H2B | Tsp_07127 |
| E5RY73 | 12 | K | Histone H2B | Tsp_07127 |
| E5RY73 | 15 | K | Histone H2B | Tsp_07127 |
| E5RY73 | 18 | K | Histone H2B | Tsp_07127 |
| E5RY73 | 21 | K | Histone H2B | Tsp_07127 |
| E5RY73 | 106 | K | Histone H2B | Tsp_07127 |
| E5RZG1 | 14 | K | Histone H2B | Tsp_07554 |
| E5RZG1 | 17 | K | Histone H2B | Tsp_07554 |
| E5RZG1 | 102 | K | Histone H2B | Tsp_07554 |
| **H3** | | | | |
| E5SEU4 | 10 | K | Histone H3 | Tsp_02260 |
| E5SEU4 | 15 | K | Histone H3 | Tsp_02260 |
| E5SEU4 | 19 | K | Histone H3 | Tsp_02260 |
| E5SEU4 | 24 | K | Histone H3 | Tsp_02260 |
| E5SEU4 | 28 | K | Histone H3 | Tsp_02260 |
| E5SEU4 | 37 | K | Histone H3 | Tsp_02260 |
| E5SEU4 | 75 | K | Histone H3 | Tsp_02260 |
| E5SEU4 | 98 | K | Histone H3 | Tsp_02260 |
| **H4** | | | | |
| E5RY75 | 6 | K | Histone H4 | Tsp_07129 |
| E5RY75 | 9 | K | Histone H4 | Tsp_07129 |
| E5RY75 | 13 | K | Histone H4 | Tsp_07129 |
| E5RY75 | 17 | K | Histone H4 | Tsp_07129 |
| E5RY75 | 60 | K | Histone H4 | Tsp_07129 |
| E5RY75 | 78 | K | Histone H4 | Tsp_07129 |
| E5RY75 | 6 | K | Histone H4 | Tsp_07129 |
| E5RY75 | 9 | K | Histone H4 | Tsp_07129 |
| E5RY75 | 13 | K | Histone H4 | Tsp_07129 |
| E5RY75 | 17 | K | Histone H4 | Tsp_07129 |
| E5RY75 | 60 | K | Histone H4 | Tsp_07129 |
| E5RY75 | 78 | K | Histone H4 | Tsp_07129 |
| E5RY75 | 92 | K | Histone H4 | Tsp_07129 |
