# Supplementary material for: Comprehensive Proteomic Analysis of Lysine Acetylation in the Foodborne Pathogen Trichinella spiralis
Source: Front Microbiol. 2018 Jan 11;8:2674. doi: 10.3389/fmicb.2017.02674 (PMC5768625; doi:10.3389/fmicb.2017.02674)
Supplement: Supplementary file 7 [file Presentation_1.PDF]

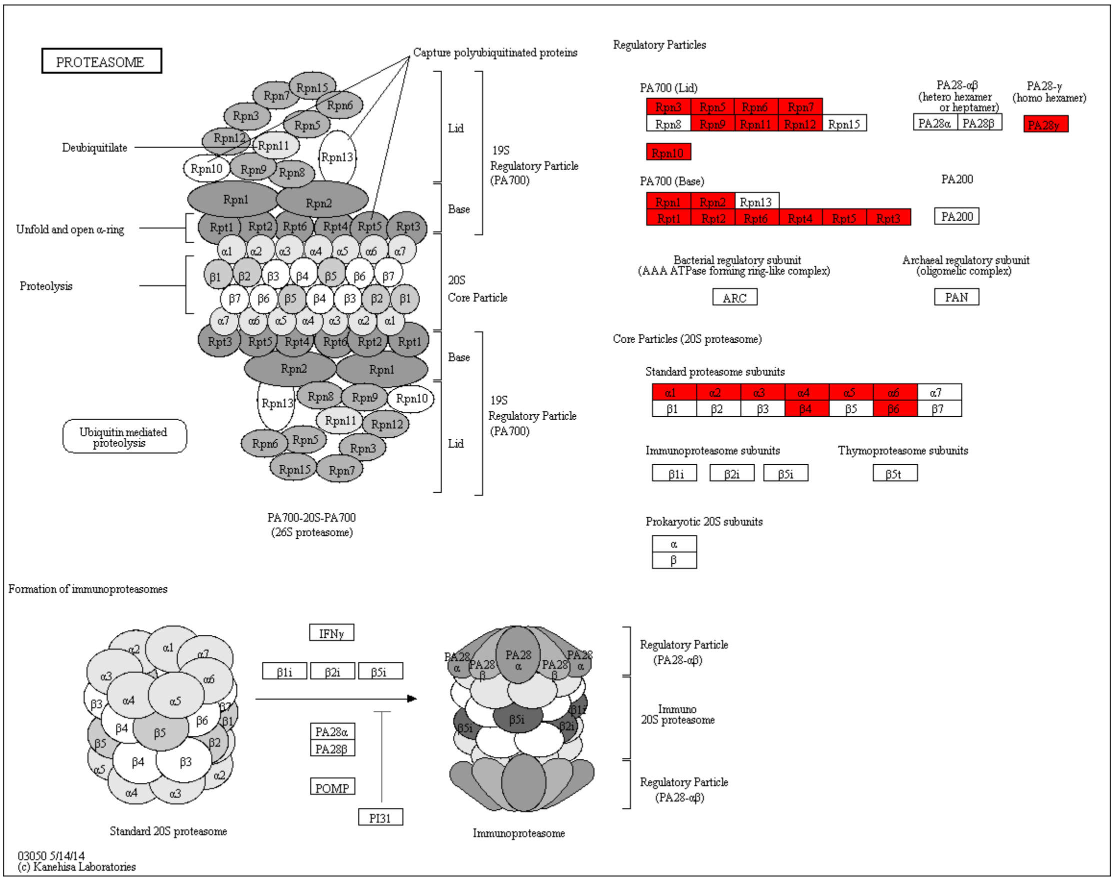

Supplementary Figure S1. Lysine-acetylated proteins involved in proteasome system of *T. spiralis*. The identified acetylated proteins are marked in red.

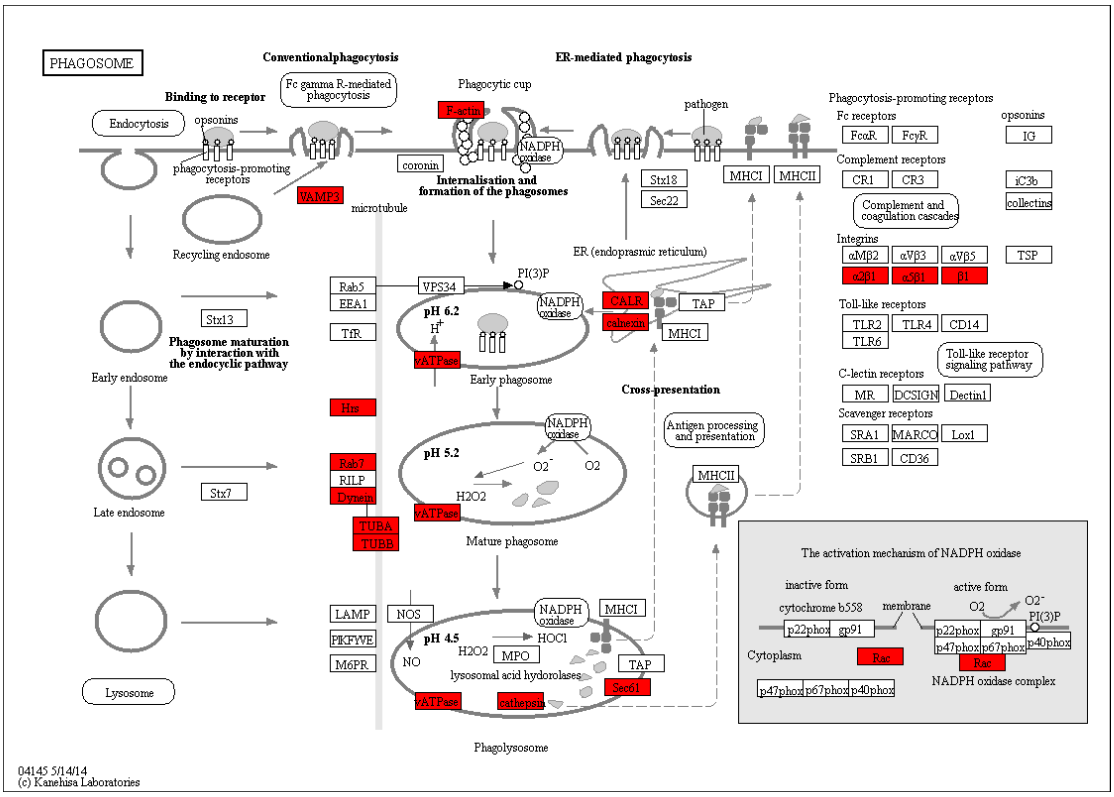

Supplementary Figure S2. Lysine-acetylated proteins involved in Phagocytosis of *T. spiralis.* The identified acetylated enzymes are marked in red.
